# System infection prevention in hospital networks – a SHEA research network survey

**DOI:** 10.1017/ash.2024.259

**Published:** 2024-09-16

**Authors:** Michael Stevens, Nkechi Emetuche, Catherine Passaretti, Graham Snyder, Rachael Snyders, Jonas Marschall

**Affiliations:** West Virginia University; SHEA; Atrium Health; UPMC/University of Pittsburgh; BJC HealthCare; Washington University School of Medicine

## Abstract

**Background:** Hospitals are increasingly consolidating into networks and integrating infection prevention (IP) into system infection prevention programs (SIPP). Very little has been published about these programs. This survey sheds light on the current state of SIPPs. **Methods:** We used the survey generator Alchemer.com for setting up the questionnaire, and tested a beta version among peers. The final version was sent out to SHEA Research Network participants in August 2023. Raw data was compiled and analyzed. **Results:** Forty institutions responded (40/104, 38%), of which 25 (63%) had SIPPs. These SIPPS reported health systems with a median of 4.5 acute care hospitals (range, 1-33); 16 SIPPS reported a median of 2 critical access hospitals (range, 1-8); 4 SIPPs reported 1-3 LTACHs, and 6 SIPPS reported a median of 1.5 nursing homes. All except 3 (88%) contained an academic center; 48% (11/23) of the U.S. based programs operate in multiple states. Four programs have been in place >20 years, four < 2 years, and the remainder a median of 8 years (range, 2-18). Physician directors also have clinical (20/25, 80%), teaching (19/25, 76%), research (15/25, 60%), antimicrobial stewardship (8/25, 32%), quality (8/25, 32%), and/or patient safety (5/25, 20%) roles. Seventeen (68%) report having a written job description. Nineteen (76%) report having an infection preventionist in a system IP director role; only 7/25 (28%) have a dedicated system IP team that operates independent of individual hospitals. Sixteen (64%) report administrative support, 10/25 (40%) have a data manager/analyst, and 4/25 (16%) include IT expert or programmer support. 15/25 (60%) report having done a formal system-wide IP needs assessment. While 16/25 (64%) have some automation in HAI surveillance (predominantly using Bugsy [Epic] or Theradoc [Premier]), while only 5/25 (20%) run fully automated surveillance. 10/25 (40%) have implemented centralized surveillance. 12/25 (48%) have “system IP policies” that are hierarchically above individual site policies. The biggest challenges appear to be gaps in 1) clear governing structure, 2) communication, 3) consistent staffing, 4) data management support, and 5) dedicated, empowered IP expert FTEs. **Conclusions:** To our knowledge, this is the first U.S. survey to explore present-day system infection prevention. In this sample of hospital networks, we found heterogeneity in the structure, staffing and resources for system IP with significant opportunities for improvement. In this era of healthcare consolidation, our findings highlight the urgent need to more clearly delineate and support system IP needs in order to enhance their functionality.

